# Tidally Forced Saltwater Intrusions might Impact the Quality of Drinking Water, the Valdivia River (40° S), Chile Estuary Case

**DOI:** 10.3390/w12092387

**Published:** 2020-08-26

**Authors:** José Garcés-Vargas, Wolfgang Schneider, Andre Pinochet, Andrea Piñones, Francisco Olguin, Daniel Brieva, Yongshan Wan

**Affiliations:** 1 Instituto de Ciencias Marinas y Limnológicas, Universidad Austral de Chile, Valdivia 5090000, Chile; 2 Centro FONDAP de Investigación en Dinámica de Ecosistemas Marinos de Altas Latitudes (IDEAL), Valdivia 5090000, Chile; 3 Departamento de Oceanografía, Universidad de Concepción, Concepción 4070043, Chile; 4 Instituto Milenio de Oceanografía (IMO), Universidad de Concepción, Concepción Casilla 1313, Chile; 5 Centro de Investigación Oceanográfica COPAS Sur-Austral, Universidad de Concepción, Concepción 4070043, Chile; 6 Center for Environmental Measurement and Modeling, USEPA, Gulf Breeze, FL 32561, USA

**Keywords:** global change, drinking water, hydrologic flood, tidal range

## Abstract

The Valdivia River estuary (VRE) located in south-central Chile is known as one of the largest estuarine ecosystems on the Pacific coast. This research aims to determine the intra-tidal and sub-tidal variability of saline intrusions into the VRE between November 2017 and March 2019 derived from salinity sensors located along the VRE. Complementary hydrographic measurements were conducted during flood and ebb conditions of the spring and neap tides for each of the four seasons of the year along the central axis of the VRE. The results of the salinity time series showed that saline intrusions (values greater than 0.5 Practical Salinity Units) occurred ~20 km from the estuary mouth, when the total flow of the Cruces and Calle-Calle rivers (main tributaries of the estuary) was low, around 280–300 m^3^ s^−1^. During the same period, the best co-variability was observed between the saline intrusions and the mixed-semidiurnal tide and the fortnightly and monthly periods of the tide. Regression analyses indicated that salinity intrusion length (L) is best correlated to discharge (D) with a fractional power model L α D^−1/2.64^ (R^2^ = 0.88). The decreasing discharge trend, found between 2008–2019, implies that saline water intrusions would negatively impact the Valdivia’s main drinking water intake during the low rainfall season under future climate conditions.

## 1. Introduction

In positive estuaries, the seawater propagates over the bottom of the river mouth, due to the difference in density between the upper fresh and lower haline water and moves upstream against the river flow. A more (less) extended seawater intrusion occurs when the river flow is weak (strong) and then reaches several tens of (a few) kilometers into the river [1]. During times of minimum river discharge, the length of seawater intrusion can negatively affect the quality of water used for domestic, agricultural, industrial, and other uses. For example, under future scenarios of sea level rise, enhanced salinity intrusions in the estuarine system along the coastal zone of Bangladesh is projected to impact the farming industry by substantially reducing yields [2]. Likewise, the availability of water supply will be reduced for cities such as Macau, Zhongshan, and Zhuhai in Southern China [3,4]. In addition, changes in the length of the saltwater intrusion also produce changes in estuarine hydrodynamics, i.e., in the circulation and the stratification of the water column, which control horizontal and vertical movement of the inhabitant species. Increase in salinity of estuarine systems also serves as a key driver of saltwater intrusion into unconfined coastal aquifers, inserting further stress on limited freshwater resources in coastal zones. This issue can become especially pronounced under future climate conditions due to sea level rise, reduced freshwater inflow, or increased demand of freshwater resources. Thus, delineating the salinity structure in estuarine systems will help in setting accurate boundary conditions for both a semi-analytical solution of the Henry saltwater intrusion problem [5] and numerical models of density-dependent groundwater flow [6].

The intrusion of seawater is frequently controlled by the tides and the degree of river flow, and to a lesser extent by the height and period of the swell, whose impact is limited to the area near the river mouth [7]. The strength of winds can play an important role during shorter periods of time (less than a week) [8]. The interplay among these controlling forces changes in time and space with strong intra-tidal and sub-tidal variability. Modulations of mixed predominantly semi-diurnal tides ought to be observed in the former category, and variations of low-frequency tidal oscillations with periods of 13–15 and 27–31 days might occur owing to variability of the winds, atmospheric pressure, floods related to severe storms, rapid snow-melt events, and seasonal changes in river discharge [9]. However, when the tidal signal in salinity is averaged daily, freshwater discharges are possibly the force that most impacts salinities [10].

In the present investigation, we analyzed the intrusion of saline water in one of the largest estuary systems of the Southeast Pacific coastal zone [11], the Valdivia River estuary (VRE), located in south-central Chile. Although some studies established relationships between saltwater intrusions and river flow [12,13], these relationships have not been quantified and the hydrographic measurements did not consider neap tide periods. In addition, their samples were taken four times a year, with each only for 1~2 days, which only allowed for the evaluation of seasonal changes.

**Figure 1.**
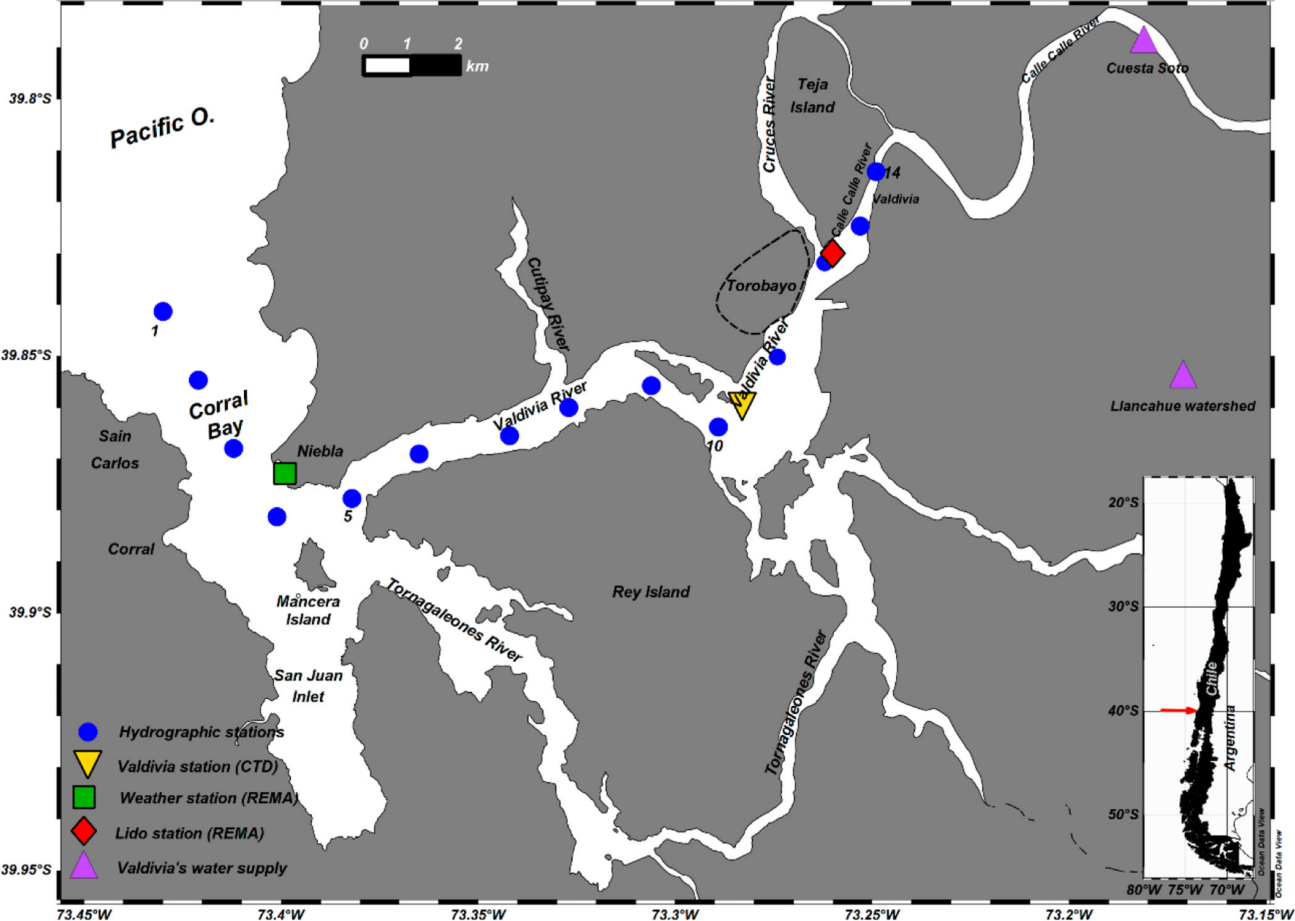
Map of the Valdivia River estuary (VRE). Right inset shows location of study area.

The dynamics of saltwater intrusion may also be strongly affected by periods of drought (reduction of precipitation and river run off) or flood. A severe drought struck the region of central and southern Chile in 2015. As a consequence, the supplier of drinking water for Valdivia city had to pump almost all of its original matrix supply during that year, instead of from the usual fresh water source in the Llancahue watershed (6 km to the southeast of Valdivia City, Figure 1), i.e., from the Calle-Calle River, where saltwater intrusion occurred and which immediately triggered massive complaints by the users of drinking water concerning a high chloride concentration and bad flavor [14]. Thus, a better understanding of the variability of the extent of saline intrusion would have helped to avoid the problems caused by pollution and the bad flavor of drinking water.

The objective of this study was to determine the extent of the saline water intrusions along the central axis of the VRE by means of hydrographic measurements during ebb and flood tides and during spring and neap tide conditions in four seasons of the year, and to establish the intra-tidal and sub-tidal variability of saline intrusions from bottom-mounted sensors installed along the Valdivia estuary. Our analysis showed that the saltwater intrusions occurred ~20 km from the Valdivia estuary mouth, when the discharges were below the threshold of 280–300 m^3^ s^−1^. During low discharges, salinities upstream varied with the mixed-semidiurnal tide and the spring–neap and apogee–perigee cycles. The best regression model showed that when the discharge is less than 74 m^3^ s^−1^, the salinity intrusion reaches the Valdivia city water intake.

## 2. Materials and Methods

### 2.1. Study Area

The VRE is located close to Valdivia city (39°49′22″ S, 73°15′0.0″ W) in south-central Chile (Figure 1). The VRE is governed by a gravitational circulation and hosts a very productive marine ecosystem. A large number of economic and recreational activities related to artisanal fisheries, aquaculture, transport, and tourism have developed in and around this estuary. Nevertheless, this estuarine system is still less exploited than the estuaries of central Chile [15] and the fjords of Chilean Patagonia, which in recent years were targeted by commercial projects to develop hydroelectricity, tourism, and aquaculture [16]. The VRE includes Corral Bay at its mouth, and inland the San Juan Inlet, which is separated from the former bay by the Mancera Island, and the rivers Valdivia and Tornagaleones. The Valdivia River is fed by the rivers Calle-Calle from the east and Cruces and Cutipay from the north, hosting one of the most important wetland systems of Chile [17]. The Valdivia river flows into Corral Bay, after traveling just 15 km where the VRE reaches its maximum depths (~25 m) [18]. The Tornagaleones River enters Corral Bay from the southeast of Mancera Island. The flow of the Calle-Calle and Cruces Rivers is in synchrony and both represent the most important components of the VRE. The sum of their annual average discharge is ~670 m^3^ s^−1^ being maximal in austral winter (~1500 m^3^ s^−1^ in July) and minimal in austral summer (~210 m^3^ s^−1^ in March) [13,19]. Tides exhibit semi-diurnal phases in Corral Bay with a mean tidal height range of 0.8 m but vary from 1.48 m during spring tides to 0.53 m during neap tides [20]. Thus, the estuary is classified as microtidal.

### 2.2. Documented Seawater Intrusion in the VRE

Up to now, the most comprehensive study on the VRE was presented by [12]. The authors determined the carrying capacity of this ecosystem in order to establish the potential growth of aquaculture activities. In the study of [12] to characterize the hydrodynamic environment, current and hydrography measurements were performed during 1 or 2 days within four seasons between 2000 and 2001. However, neither stratification of the water column nor saline intrusion were keenly analyzed. More recently, the hydrographic data obtained by [12] corresponding to the middle part of the estuary of the Valdivia River and the mouth of the Corral Bay were examined together with tides, river flow, wind, and solar radiation to delineate the thermal and haline structure of this part of the VRE [13]. This study established that the VRE varied seasonally and behaved like a salt-wedge in austral winter and austral spring due to a large water transport by the rivers. In these seasons, the salt intrusion length (defined as an upstream distance of the isohaline close to zero) failed to arrive at the mouth of the Cutipay River (~10 km from the mouth of the Corral Bay), due to large river discharges into the basin. In austral summer and autumn, the estuary behaved as partially mixed, owing to a lesser river discharge. Major changes in intrusion length and stratification between flood tide and ebb tide were observed in April, when the river transport was at its minimum. Salt intrusion extended all the way into the Calle-Calle River (~22 km from the mouth of Corral Bay) during flood tides, and the estuary behaved as partially mixed. By contrast, during ebb tide, the intrusion length only arrived near the mouth of the Cutipay River; the estuary was well mixed based on salinity profiles. Therefore, in the VRE, saline intrusion and vertical stratification varied seasonally, shaped primarily by the tides and river discharge. Maximum intrusion length and reduced water column stratification were attained when river discharge was lower (summer–autumn), being opposite when river discharge was larger (winter–spring).

The measurements by [12] were obtained only during good weather conditions and did not include neap tide conditions. One would suppose that the measured saltwater intrusion length was shorter (larger) during neap (spring) tide because tidal currents were weaker (stronger) and therefore would imply less (more) salinity advection. If the stratification is either very strong or very weak and there are no large neap–spring changes in stratification, these expectations would be fulfilled [9]. However, in intermediate stratification conditions, which occurred during much of the year, stronger (weaker) vertical mixing during spring (neap) tides tended to reduce (increases) density stratification. Thus, during spring (neap) periods stronger (weaker) vertical mixing allows higher (lower) dissipation throughout the water column and inhibits (facilitates) saltwater intrusion, as has been observed in the Columbia River estuary for low river discharge conditions [21]. In the VRE, the differences in the range of neap–spring tides are almost one meter (from 34% less than the average range up to 85% over the average range) and stratification was only described during spring tides. Therefore, it is important to understand in detail whether stratification and turbulent mixing take place during neap tides in different seasons of the year and to know how far the intrusion of saline water extends.

Although winds play a less important role in driving saltwater intrusions into the VRE [13], they can become important under certain conditions. Winds could break stratification of the water column when competing with buoyancy, which in turn is controlled mainly by salinity during certain periods. Winds and buoyancy could play a dominant role in the mean circulation [8] and consequently vary the extension of the saline intrusion. Therefore, a knowledge of the local winds is crucial, taking into account that satellite-measured winds were employed in the work of [13], where the nearest grid was ~50 km from the mouth of Corral Bay and most likely very different from the local wind.

The wetland located in Cruces River, called Carlos Anwandter Nature Sanctuary, underwent a process of rapid environmental degradation in 2004–2005, where black-necked swans (one of the largest breeding colonies in South America) died or migrated as well as other water bird species [17]. This collapse was principally caused by pollution from an installed pulp mill located upstream [22] and was sentenced for environmental damage [23]. However, the study conducted by [22] was controversial and pointed out that there was no information of saltwater intrusion either in any tidal category and season nor detailed studies of the extent of the tidal zone.

### 2.3. Data

#### 2.3.1. Moored Sensors and Hydrographic Stations along the VRE

A mooring was installed at Station Lido close to the mouth of the Cruces River, just south of Valdivia City (Figure 1, red diamond). Salinity was registered at the surface and at the bottom of the river (3 m depth) as well as at the water level. Data were recorded every 10 min between November 2017 and May 2019 and were provided by the Red de Monitoreo Ambiental-Valdivia project.

Farther downstream of Valdivia River a conductivity, temperature, and depth (CTD) instrument was installed on the bottom of the river (8 m depth) and registered from November 2017 to July 2018, here referred to as Station Valdivia (Figure 1, yellow triangle). The conductivity sensor of the CTD instrument was unfortunately configured for the range 13~63 mS cm^−1^, meaning that only salinities above about 9 Practical Salinity Units (PSU), depending on the river’s bottom temperature, were measured.

Short-term cruises were conducted for each of the four seasons between November 2017 and August 2018 (see Table 1). Hydrographic measurements were carried out along the central axes of the VRE. CTD profiles were measured at 14 roughly equally spaced stations along the VRE (Figure 1) during both spring and neap tide periods of all seasons. The length of the transect was around 23 km and spanned from the end of the Calle-Calle river far into the open ocean beyond Corral Bay. The transect was first sampled from the inner station (Station 14) to the outer station (Station 1) during ebb (flood) tides and the return trips were timed to coincide with flood (ebb) tides. The profiles were taken with a horizontally configured and mounted pumped Seabird 25 CTD instrument, which allowed a slow sampling from close to the surface to close to the bottom. The raw data were processed following the manufacturer’s guidelines and were averaged to a vertical resolution of 0.25 m.

#### 2.3.2. River Runoff

The Chilean “Dirección General de Aguas” provided, for the period of November 2017 to May 2019, the daily mean and daily maximum estimates of river runoff for the rivers Calle-Calle (Pupunahue station, 39°48′16″ S, 72°54′9″ W) and Cruces (Rucaco station, 39°33′ S, 72°54′ W), which constitute the main sources of fresh water flow into the VRE. The Pupunahue and Rucaco stations are located ~42 and ~68 km east of Corral Bay, respectively.

### 2.4. Methods

#### 2.4.1. Tidal Analysis

The tidal analysis was split in two: (1) intra-tidal variability (tidal periods from 12–25 h) and (2) sub-tidal variability (tidal periods larger than 1 day). The tidal harmonics were extracted from the water level records of the Valdivia and Lido stations using the unified tidal analysis and prediction (UTIDE) MATLAB functions [24]. The astronomic tidal record at these stations could then be reconstructed by means of these harmonics. For the reconstruction of the sum of the intra-tidal components, based on an hourly resolution, only the harmonics that exhibited a signal-to-noise ratio larger than 2 were included. In order to isolate the sub-tidal tides, the average of the two high tides minus the average of the two low tides of a lunar day (24 h 50 min) of the reconstructed astronomic tidal record was computed for each lunar day (mean daily tidal range). This way the intra-tidal components were suppressed. This time series based on lunar days was then interpolated on intervals corresponding to a solar day.

#### 2.4.2. Cross Wavelet Transformation

A wavelet transform of a time series allows finding localized sporadic periodicities and their associated phases therein. This method consists in applying a wavelet (a function with zero mean that is localized in both frequency and time) as a band-pass filter to the time series. When it is desirable to examine two time series together that are expected to be linked in some way, a cross wavelet transform is a powerful tool. The cross wavelet transformation method allows analyzing time-frequency domain and phase relationships in time between two time series [25]. First, wavelet transforms are computed and second their common power and relative phase in time-frequency space are obtained. This method thus describes the similarity and differences between the wavelet transforms of two independently obtained time series. Here, the first time series is the mean daily tidal range at Station Lido (Section 2.4.1) and the second one is the bottom salinity (Section 2.3.1).

#### 2.4.3. Determination of the Extent of Saltwater Intrusion

Sections of salinity versus depth were constructed from the profiles of each hydrographic transect carried out during spring and neap tide periods and for all four seasons along the central axis of the VRE from the outermost to the innermost station (Section 2.3.1). The ebb and flood tide sections for each campaign were averaged. The distance of the saltwater intrusions was obtained via the averaged salinity sections; in total, eight sections were built. The extent of saltwater intrusion into the VRE was defined as the distance from the outermost station (Station 1) to the location where the salinity contour near the bottom reached 1 PSU.

#### 2.4.4. Relationship between Saltwater Intrusion and River Discharge

To establish the relationship between river discharge(s) and saline intrusions into the VRE, some of the discharge measures used by [26] were evaluated. For the discharge measurements, the Calle-Calle River was considered the main contributor, the river with the maximum flow in the VRE [13], and the Cruces River as a secondary one. The different discharge measures applied are detailed below.

Hydrologic flood method: chooses the maximum average daily flow rate of the main discharge (Calle-Calle River), within a 15-day window prior to sampling. The 15-day threshold was chosen because, similar to [26], we observed that R^2^ values decreased dramatically after that threshold, as in the other methods used.

Complete hydrologic flood method: like method 1, but the discharge of Cruces River was added.

Complete value method: chooses the average flow of the sum of both rivers out of a 15-day window before sampling that yielded the best fit; here the seventh day before sampling was chosen.

Averaging method: chooses the average daily flow of the Calle-Calle River within a 15-day window before sampling.

Complete averaging method: like method 4, but the discharge of Cruces River was added.

In order to better interpret the relationship between flow rate(s) and saltwater intrusion in the VRE, we performed linear, power, and exponential regression modeling. The coefficient of determination R^2^ of each model and discharge measure was used to quantify the relationships between discharges and saline intrusions.

## 3. Results

### 3.1. Extension of Saline Water Intrusions along the Central Axis of the VRE

**Figure 2.**
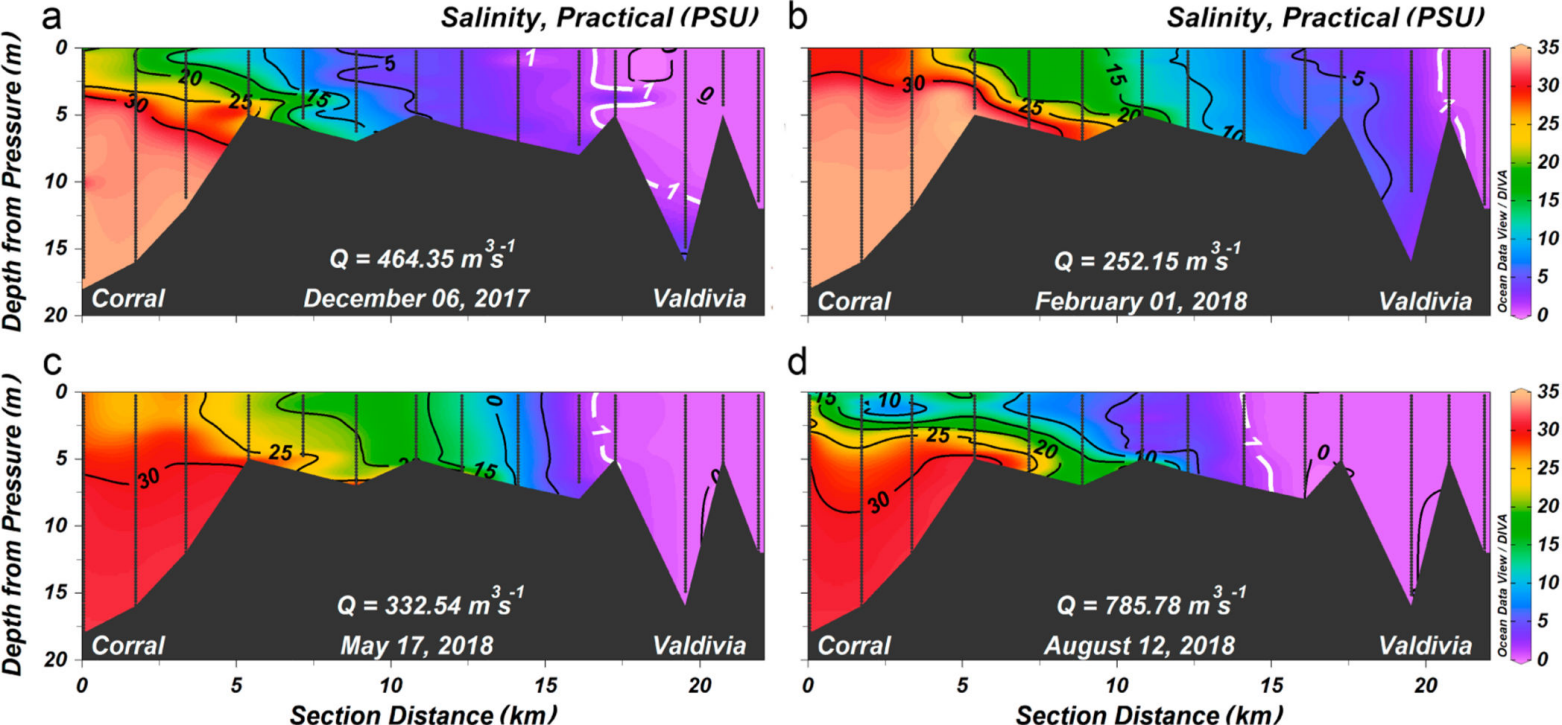
Mean salinity (ebb and flood tides) along the central axis of the VRE during spring tides for different seasons of the year. (**a**) Austral spring, 6 December 2017. (**b**) Austral summer, 1 February 2018. (**c**) Austral fall, 17 May 2018 and (**d**) Austral winter, 12 August 2018. The vertical gray lines in each panel mark the positions where the CTD profiles were taken. The black contour lines are isohalines in intervals of 5 PSU. The 1 PSU isohaline (white contour) was employed to determine the extent of saltwater intrusion; *Q* is the sum of the average flows of the Calle-Calle and Cruces rivers 7 days prior to sampling.

Sections of salinity along the VRE during spring tide sampled in austral spring 2017, australsummer 2018, austral fall 2018, and austral winter 2018 are displayed in Figure 2 (to identify the locations of the individual stations please refer to Figure 1). Each section was sampled during ebb and flood tides from the innermost to the outermost station; both sections were averaged. The combined river discharges (7 days prior to sampling) were different in all seasons sampled: from austral spring to winter 464, 252, 332, and 785 m^3^ s^−1^. The intrusion of saltwater (a threshold of 1 PSU was chosen) was indirectly proportional to the strength of river flow, i.e., the lesser the flow, the farther the intrusion extended. In austral summer, on 1 February 2018, when the river discharges were at their minimum, saltwater was detected 22 km upstream from the estuary entrance. By contrast, in austral winter (12 August 2018), when the river flow was high, saltwater reached only 15 km into the Valdivia River. During austral spring and fall, both flow rate and intrusion were at intermediate levels. Saltwater intruded somewhat farther into the river along the bottom in all sections, and in that position there was little stratification of the water column.

In order to statistically relate the extension of the saltwater intrusions to the river discharges of the Calle-Calle and Cruces rivers, linear, power, and exponential regression analyses were carried out considering five discharge measures (see Data and Methods). Saline intrusion estimates were obtained from the seasonal hydrographic sections during spring and neap tides (Table 1); ebb and flood tide hydrographic transects were averaged. The regression results are presented in Table 2 for all combinations of regression models and discharge measures. The *R*^2^ values ranged from 0.53 to 0.88. The power regression models, in general, yielded the best results, followed by the exponential and linear models. The discharge measures, complete hydrologic flood, complete value, and complete averaging methods, which included the discharge of both rivers, generated higher correlations in all regression models. In all regression models, the average flow of the sum of both rivers on the seventh day before sampling (complete value method) yielded the highest *R*^2^ values: 0.88, 0.77, and 0.72 for the power, exponential, and linear regression models, respectively.

**Figure 3.**
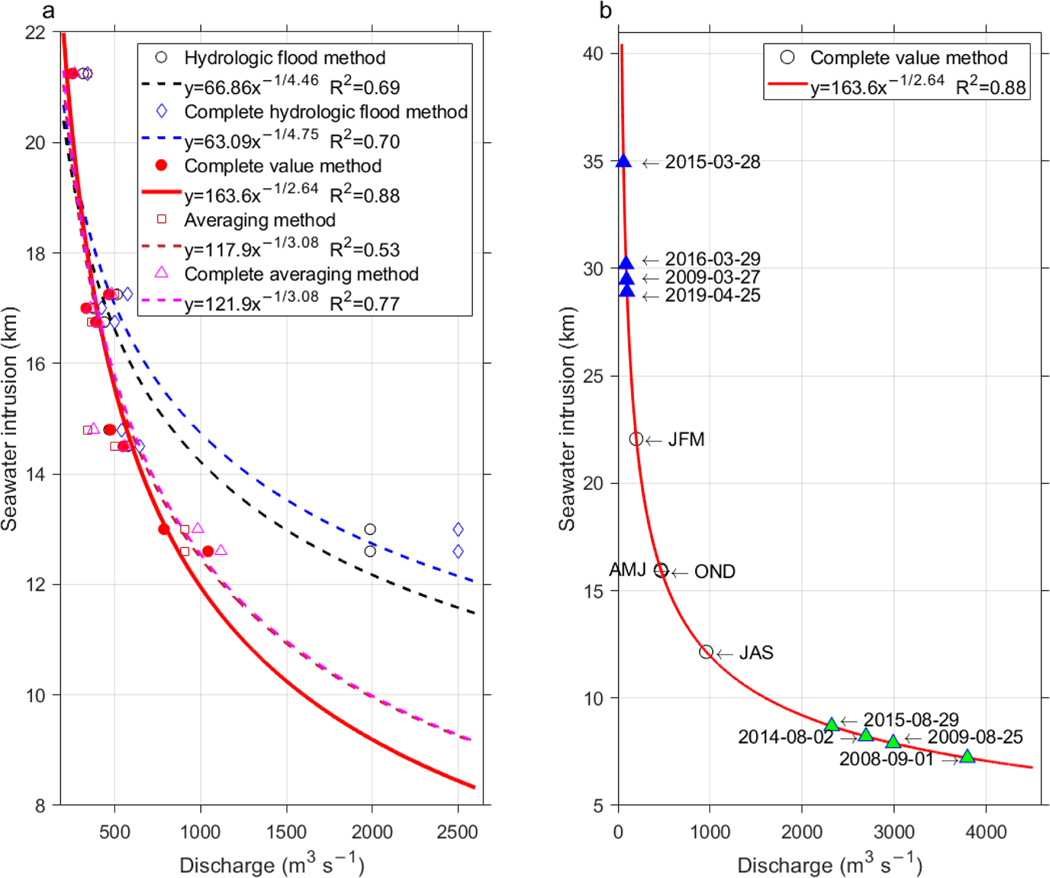
(**a**) Regression curves between the different measures of discharge(s) from the river(s) and saline water intrusions (average of ebb and flood type) in the VRE for the four seasons of the year under conditions of spring and neap tides applying power regression models. (**b**) The power regression curve that was obtained between the complete value method discharges and saltwater intrusions with a R^2^ score of 0.88 extended to a discharge range of 0–4500 m^3^ s^−1^. Long-term (2008–2019) seasonal averages of river discharges for January–March (JFM), austral summer, April–June (AMJ), austral autumn, July–September (JAS), austral winter, and October–December (OND), austral spring, are shown as circles. Extreme flow values are shown as triangles (minimums in blue and maximums in green).

The curves of the power regression model used to fit saltwater intrusion to flow rates by applying different methods to estimate the flow are shown in Figure 3a. When discharges were low (<600 m^3^ s^−1^), the flow estimates for the different methods used were not widely dispersed and in general the model fit to determine saltwater intrusion was good. However, when discharges increased, the dispersion of flows according to different measures was wide. This was especially noticeable for the austral winter–spring tide sample (second to last seawater intrusion), where for example for the complete value method (7 days prior to sample) and complete hydrologic flood method (maximum flow rate within a 15-day window prior to sampling) the flow values were 786 and 2502 m^3^ s^−1^, respectively. Persistent high flow rates would, however, not allow a saltwater intrusion of 13 km (see Section 3.3), and in this case R^2^ is only 0.7; thus the complete hydrologic flood method was rejected. The power regression model in which the complete value method discharge measure was applied yielded the best fit and explained 88% of the observed variance: intrusion (km) = 163.6; discharges (m^3^ s^−1^)^−1/2.64^; it was thus chosen based on the highest squared correlation.

During the particular times in which the hydrographic transects were performed, the combined river discharges 7 days prior to sampling happened to be in the range of 252–786 m^3^ s^−1^. Long-term (2008–2019) seasonal averages of river discharges were for January–March, austral summer (198 m^3^ s^−1^), April–June, austral autumn (470 m^3^ s^−1^), July–September, austral winter (957 m^3^ s^−1^), and October–December, austral spring (465 m^3^ s^−1^), which puts our seasonal snapshots (Figure 2) in the range of average or less than average conditions. When applying the best regression fit to these flow rates, saltwater intrusion resulted to be between 12 (austral winter) and 22 (austral summer) km (Figure 3b), which was in good agreement with the in situ observations, although the austral winter cruise registered an intrusion of 15 km due to the lesser than average long-term flow rate. The austral summer cruise did not coincide with annual minimum river discharges, which usually occurred by the end of March (see Section 3.3); therefore, intrusions are expected to reach beyond those 22 km as estimated for 1 February 2018. Between 2008 and 2019, minimal (maximal) discharges were lesser (higher) than 60 (3800) m^3^ s^−1^ on 28 March 2015 (1 September 2008) with an estimated saltwater intrusion of 35 (7) km (Figure 3b).

### 3.2. Harmonic Analysis of Tides

To bridge the gap between the seasonal hydrographic samplings and to enhance the time of observations, a mooring was installed just south of Valdivia City, labeled here Lido station; surface and bottom salinity and river level were registered from November 2017 to May 2019, and in Valdivia station somewhat farther downstream on the Valdivia river from November 2017 to July 2018 (see Data and Methods). The main harmonic constituents that made up the astronomical tidal wave at the Lido and Valdivia stations were the M2, K1, S2, O1, and N2 constituents from highest to lesser significance, and they explained 85.75% and 96.81% of the observed water level time series, respectively (Table 3). The amplitudes of the main components of the tides were similar in both stations, adding up to 0.74 and 0.77 m for the Lido and Valdivia stations, respectively. The astronomical reconstructions of both water level records were out of phase by 20 min (Sampling interval was 10 min) and arrived first at Station Valdivia as expected. The sum of the amplitudes of the M2 and S2 constituents, which determine the bi-weekly variability in water level height (14.8 days, spring–neap tide cycle) were more important than the sum of the amplitudes of the M2 and N2 constituents, which define the 27.6-day perigee–apogee cycle in water level height.

The factor *F* relates the sum of the amplitudes of the two most important principal diurnal harmonic constituents (K1 and O1) to the two main principal semi-diurnal ones (M2 and S2). For the Lido and Valdivia stations, *F* resulted in 0.51 and 0.48, respectively, thus classifying both tidal records as mixed tides predominantly of the semi-diurnal type [27].

### 3.3. Sub-Tidal Variability at Station Lido

**Figure 4.**
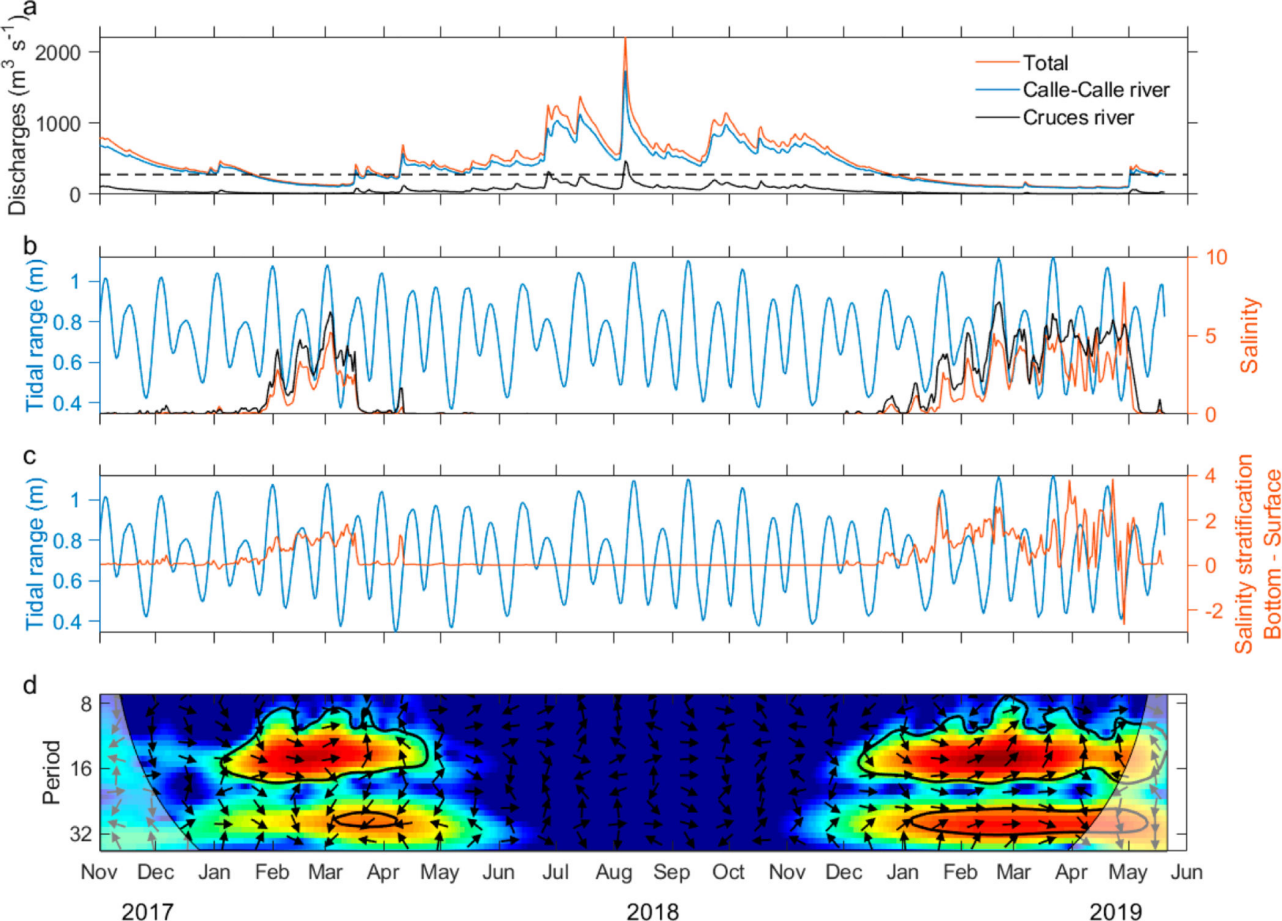
Daily time series from November 2017 to May 2019. (**a**) Discharges of the rivers Calle-Calle (blue line), Cruces (black line), and the sum of both rivers (red line); (**b**) mean daily tidal range (blue line), bottom salinity (3 m, black line), and surface salinity (red line) at the Lido station; (**c**) mean daily tidal range (blue line) and salinity stratification (bottom–surface, red line) at the Lido station; and (**d**) cross wavelet transformation between bottom salinity and tidal range at the Lido station. Common power, blue = low, red = high; the arrows pointing to the right indicate that both time series were in phase and vice versa; the 5% significance level against red noise is shown as a thick contour. In (**a**) the dotted black line shows the value of 280 m^3^ s^−1^.

The impact of tidal range variability due to spring–neap and perigee–apogee cycles, plus combined low river flow on salinity intrusion and stratification, is shown in Figure 4. This figure illustrates the impact of tidal range variability owing to spring–neap and perigee–apogee cycles and combined low river flow on salinity intrusion and stratification. By far the largest contributor to river flow in the VRE is the Calle-Calle River, which surpasses the Cruces River’s contribution by about seven times (Figure 4a). The river’s discharge exhibited a seasonal distinction, with the highest flows from April to November 2018, reaching maximum runoffs in August 2018 (>2200 m^3^ s^−1^) and the lowest between Decembers to May 2017–2018 and 2018–2019 (<500 m^3^ s^−1^). A year-to-year difference could be observed, with a lower average flow between November–May in 2018–2019 compared to 2017–2018. The tidal range properly preserved the fluctuations typical for bi-weekly and monthly variability, namely spring–neap tide and perigee–apogee tide (Figure 4b,c). Saltwater intrusions of up to 5 PSU took place during both periods of low river discharges, in November–May, and only then, but were more intense and longer-lasting during 2018–2019 than during 2017–2018, consistent with the lower river flow in the former period. In general, these saline water intrusions occurred when the total river flow was less than 280–300 m^3^ s^−1^ and were approximately in phase with the spring and neap tides, with greater increases in salinity and stratification during spring tides. Modulations of monthly variability were best observed in the 2018–2019 period, which was longer. Therefore, it was observed that both cycles, spring–neap and perigee–apogee, modified salinity when river discharges were low, producing higher salinities during spring tides.

The cross wavelet transformation measures the similarity between two time series, here the tidal range and bottom salinity. Common power during the time of observation is shown in color in Figure 4d. The phase relation between both time series during the time is indicated by the direction of the arrows. The only periods of high common power were from January to May, the times of the lowest river discharges in 2018 and 2019 as well. High similarity in power during these periods was centered on the biweekly and monthly periods, mostly in phase (arrows pointing to the right). The signal was stronger in the bi-weekly period in both occasions. Common high power was observed for a longer time in 2019, in agreement with a longer phase of low river flow.

### 3.4. River Discharge and Intra-Tidal Constituents Control Salinity at the Lido and Valdivia Stations

**Figure 5.**
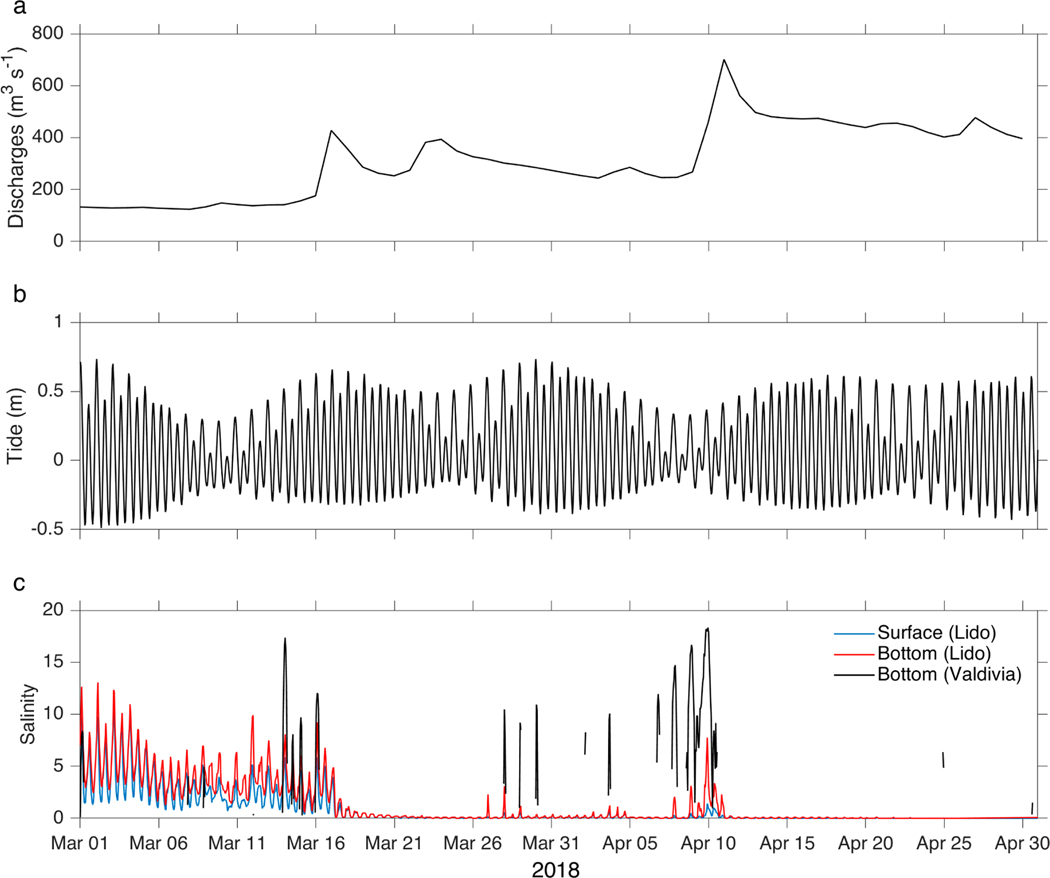
Hourly time series during March and April 2018. (**a**) Sum of the daily flows of the Calle-Calle and Cruces rivers; (**b**) reconstruction of the hourly astronomical tide at the Lido station; and (**c**) bottom (3 m, red line) and surface (blue line) hourly salinity at Lido, and bottom (8 m, black line) hourly salinity at Valdivia.

Hourly time series of the combined discharges of the Calle-Calle and Cruces rivers, the reconstructed tidal record, surface and bottom salinity at the Lido station, and bottom salinity at the Valdivia station, all for March and April 2018, are depicted in Figure 5. The control that the height and phase of the tides have on salinity during conditions of low river discharge is effectively revealed in this particular time window. During times of low river discharges, around 150 m^3^ s^−1^, during the first 18 days of March (Figure 5a), a well-established synchrony between the height of high and low tides and salinity (bottom and surface as well) was observed (Figure 5b,c). Salinity oscillated with the tides between 1–12 PSU, where the bottom salinities exceeded those of the surface, thus giving rise to periodically occurring salinity intrusions related directly to tidal periods. At the Valdivia station, which is situated somewhat closer (11 km from hydrographic station 4, Figure 1) to Corral Bay, salinities even reached more than 15 PSU close to high tides, although this record presented many gaps due to limitations of the conductivity sensors’ range (see Data and Methods). When the combined discharges more than doubled abruptly, on March 17, to values above 400 m^3^ s^−1^, the salinity fluctuations related to the height of the tides drastically reduced, to values of less than 0.5 PSU at the Lido station. The combined river flow dropped again between 21 March and 10 April; however, it did not drop to the levels of beginning of March but nevertheless to around 300 m^3^ s^−1^. Only from time to time a few weak bottom salinity peaks were registered, of up to 2 (10) PSU at the Lido and Valdivia stations. The magnitude of these minor peaks increased around 10 April to the neap tide levels of the beginning of March. River discharges then increased with the onset of austral fall to well above 400 m^3^ s^−1^, thus shutting down saltwater intrusions at its part of the VRE.

The effect of the tide on the saline intrusions at the Lido and Valdivia stations could be better understood by carrying out a more detailed analysis between 13 and 16 March 2018, during nearly spring tide with river discharges of less than 200 m^3^ s^−1^. Surface salinity fluctuations were in phase with the height of the tides, with values ranging between about 0–8 PSU at the Lido station. However, at the bottom of the river maximum salinities occurred about an hour later during flood tides with slightly higher salinity, up to more than 10 PSU at Valdivia station. During flood tides, this delay caused the stratification measure (bottom–surface salinity) to increase and reached values above 2 at the Lido station, whereas the stratification decreased during ebb tides (values close to 0).

## 4. Discussion

The stratification in the VRE changes from a salt-wedge type during times of high river discharge in austral winter to weakly stratified during periods of low discharges in austral summer [13]. In this study we investigated the extent of saltwater intrusions into the VRE on intra-tidal (12–25 h) and sub–tidal (>1 day) time scales in relation to total river discharge throughout the four seasons of the year. Moored stations (Lido and Valdivia) were employed and hydrographic transects along the central axis of the Valdivia River were carried out. The quality of drinking water, which is pumped further upstream on the river in dry summers, was addressed.

### 4.1. Extension of Saline Water Intrusions along the Central Axis of the VRE

Salt transport into estuaries can occur through diffusive and/or advective processes [28]. When salt transport upstream is dominated by advective processes, it is mainly due to gravitational circulation. However, when it is dominated by diffusive processes, mixing processes are important, such as those produced by tides that occur in unidirectional net flows. An analysis of the saltwater intrusions based on the hydrographic sections with respect to river discharges indicated that the length of salinity intrusion (L) best fitted the discharge (D) applying the fractional power model L = D^−1/2.64^ (considering the discharges of both rivers 7 days before sampling). This behavior is close to the theoretical solution for a rectangular channel that would involve more advective than diffusive processes in salt transport. The regression models used to describe the relationship between discharge and saltwater intrusion always obtained a better correlation when considering the sum of both rivers, i.e., complete hydrologic flood, complete value, and complete averaging methods. This underlined the importance of always considering the discharges of both rivers.

The time series of bottom salinity at the Lido station showed that maximum salinities, and hence maximum salinity intrusions, occurred around high tides. The sampling of the hydrographic sections started in Valdivia city early in the morning during high or low tide depending on the day of sampling. When started during high (low) tide, they were sampled downstream during ebb (flood) tide and upstream during flood (ebb) tide, this way arriving in Valdivia city with the next high (low) tide. Thus, the maximum intrusion of saltwater could be approximately measured. The river discharges 7 days before the sampling campaigns ranged between ~250–800 m^3^ s^−1^ and did not include extreme low/high discharges as observed from November 2017 to May 2019 (Figure 4). Therefore, estimates for the length of saltwater intrusions at the low/high discharge ends of the regression curve are associated with a certain error. The extreme values of the daily discharges greatly undercut or exceeded the average seasonal values and occurred in March–April and August–September (Figure 3b). The model that best fitted the observations of discharge and saltwater intrusion estimated that during times of minimum daily discharges (2008 to 2019) the extent of saltwater intrusion reached more than 29 km into the estuary, that is, about 7 km beyond our most upstream hydrographic station, station 14 (Figure 1). For maximum daily discharges the extension of the saline water intrusion was less than 9 km, that is, around station 6 (Figure 1).

The extent of saline intrusion was farther upstream during spring than neap tides, which indicated that the tidal currents advected more saltwater during the former tidal phases. Similarly, in the Sebou River estuary, the largest Moroccan river, the length of saltwater intrusion reached ~46 km during spring tides and only ~12 km during neap tides (for a low river discharge of 60 m^3^ s^−1^) [29]. The far-reaching intrusion during spring tides in this Moroccan river was related to the rather high tidal range of ~2.25 m (~1–3.25 m) between neap and spring tides compared to ~0.7 m (~0.4–1.1 m) for the VRE. Observing longer saltwater intrusions during spring tides contrasts with other estuaries, where the larger saltwater intrusions occurred during neap rather than spring tides, such as the Cape Fear River estuary, North Carolina [30] or the Columbia River estuary [21]. In these estuaries the stratification of the water column was intermediate and there were huge changes in the stratification from neap to spring tides. The strong vertical mixing during spring tides reduced the density stratification, which allowed a better dissipation in the whole water column, thus inhibiting a salinity advection [21].

### 4.2. River Discharge and Tidal Constituents Control Salinity at the Lido and Valdivia Stations

During the periods when the flow of the Calle-Calle and Cruces rivers was low, the salinity intrusions at the Valdivia and Lido stations were in phase with the mixed-semidiurnal local tides—highest (lowest) salinity during high (low) tides. At the Lido station, during ebb tides, the less dense surface water moved faster than the denser bottom water, producing stratification and favoring baroclinic circulation, congruent with tidal straining [31] and perhaps important for planktonic species distribution, as has been observed in the Rhine outflow area [32]. On the contrary, during flood tides, the vertical mixing was strong and destratified the water column. Although at the Valdivia station no such effect could be seen, due to a lack of records, salinity was in phase during flood tides at the surface with bottom salinity at the Lido station. Most likely, at the Valdivia station the effect of tidal straining changed a bit since it is situated closer to the mouth of the estuary as tidal straining has been observed to depend on the position within the channel, e.g., [26]. In any case, estuarine circulation would strongly increase.

The impact sub-tidal variability had on salinity was evident during periods of low river flows at the Lido station. The increase in salinity was much more noticeable in the periods of spring than neap tides, as this was observed in the extent of saltwater intrusion discussed in the previous section. The modulations imposed by the spring–neap cycle on salinity variability have also been observed for example in the Pearl River estuary in Southern China [3] and the Cape Fear River estuary [26]. An impact of the perigee–apogee cycle on salinity was not observed in the Pearl River estuary, which most likely was related to the fact that the periods of low river flow were less than 50 days, unlike in the Cape Fear River estuary, where monthly tidal variability in salinity was notorious.

Wind can be a major force on intra- and sub-tidal scales, at times. Local winds at the meteorological station located in Niebla (Figure 1, green square) were analyzed for the months in which river discharges were low. During the summer months of the year 2019 winds were stronger and more variable than those of the year 2018 (Table 4). Although they were relatively low, many gusts over 4 m s^−1^ were observed. This could be an explanation for why seawater intrusions did not adjust as well with the sub-tidal periods and with the salinity stratification in the year 2019. The wind, instead of causing a greater intrusion from the mouth into the estuary, could have caused mixing in the water column and would force the water to overturn. This would explain the stratification index values, which were close to zero and even negative. This was much more evident during April 2019, when there were many periods when the water column was destratified and river discharges were very low (Figure 4a,c). However, it is very likely that salinity will be strongly affected at the mouth of the estuary due to greater exposure to offshore wind.

### 4.3. Implications of Saltwater Intrusions for the Provider of Drinking Water for Valdivia City

When the total flow of the Calle-Calle and Cruces rivers was lower than 280–300 m^3^ s^−1^, surface and bottom saltwater intrusions with salinities of 5–10 PSU, much higher than 0.5, were registered at the Lido station (situated at 20 km from the oceanic station 1) near the Torobayo sector (Figure 1), 0.5 being a threshold recommended as the maximum permitted for water consumption [33]. This, for example, occurred between February and April 2018 and repeated itself from January to May 2019. In these periods the saline intrusions synchronized well with the mixed-semidiurnal tide and the fortnightly and monthly tidal periods—higher (lower) tides allowed for higher (lower) salinity intrusions.

**Figure 6.**
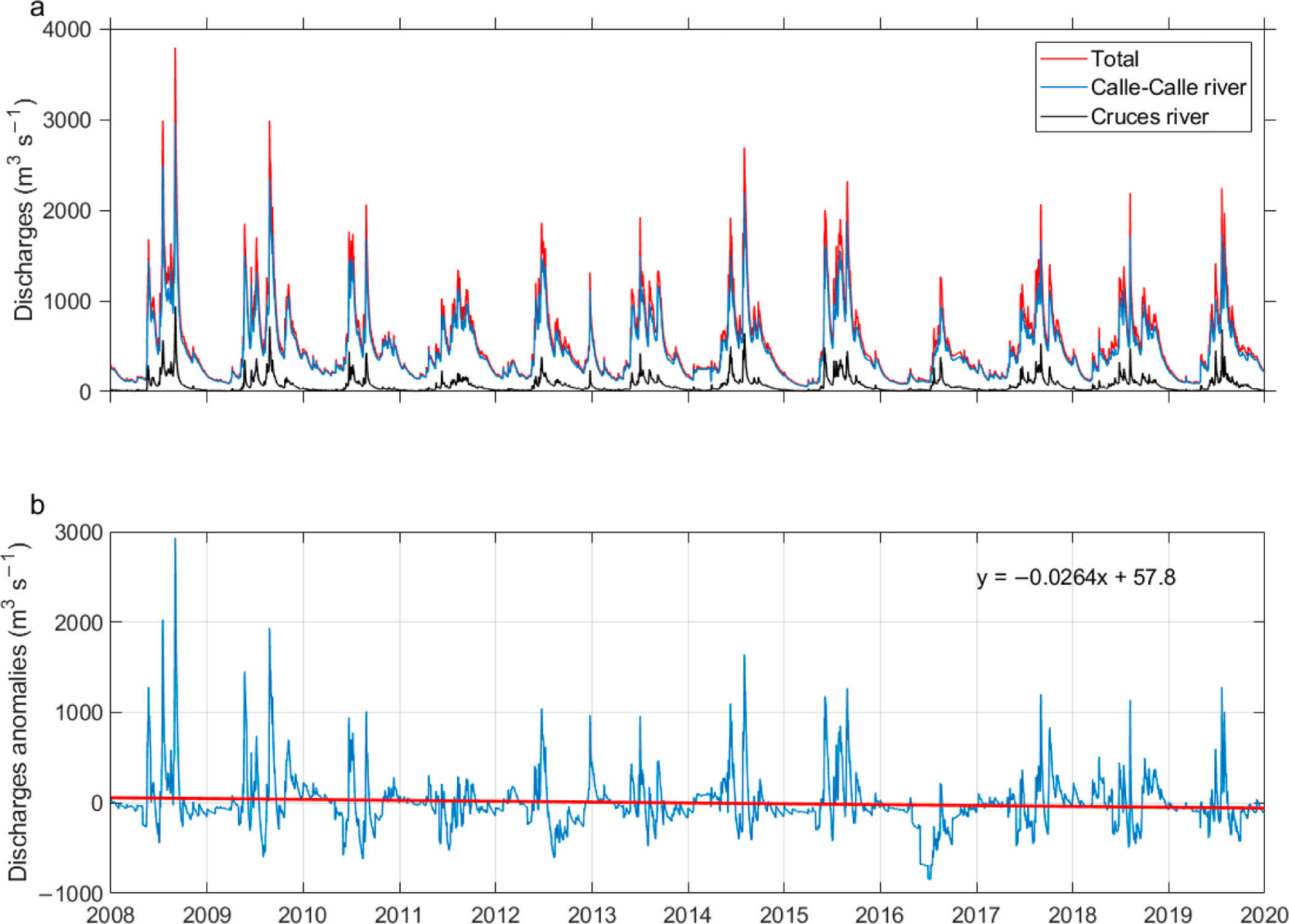
Time series of daily (**a**) river flow of the Calle-Calle (blue line) and Cruces (black line) rivers and sum of both rivers (red line) and (**b**) discharge anomalies of the sum of the Calle-Calle and Cruces rivers. The red line in (**b**) represents the linear trend from 2008 to 2019 and its mathematical representation is shown in the upper right corner. The flow data anomalies were based on the long-term daily means: 2008 and 2019.

This is a worrying fact for the city of Valdivia, since, during the dry season, the drinking water company changes a large part of its main fresh water matrix from the Yancahue watershed to the southern bank of the Calle-Calle River, Cuesta Soto, whose intake pump is located 32 km from Corral Bay (Figure 1). Thus, for example, in the austral summer of 2015, specifically March, the combined flow of the rivers was low (Figure 6a) and citizens complained about the bad flavor of the water as mentioned in the Introduction. The minimum flow value for that month was 59 m^3^ s^−1^ (Figure 3b) and according to the complete value method model the length of saltwater intrusion would extend 35 km, surpassing the alternative water collection site for the city. Thus, for the future it is expected that there could be numerous events of saline water incursions at the Cuesta Soto water catchment site and that these intrusions would extend into the austral autumn. According to this study, for salinity intrusions to reach the Cuesta Soto water intake, the flow of both rivers would have to be less than 74 m^3^ s^−1^, which happened in almost all of March 2015. This alternative source of fresh water is currently subject to saltwater intrusions; projections for the reductions of rainfall and therefore river flow for the future indicate these intrusions will continue.

A significant negative trend in annual rainfall (~−5% per decade^−1^) was observed in the region of Valdivia city, which prevailed from the 1950s to the 1990s [34]. In addition, a more recent study showed a substantial decrease in precipitation between 2003 and 2014 [19]. As precipitation is the main driver for river flow [35], we expect that the river flow rates in the VRE decreased equivalently. In fact, this is a viable scenario, as there is a linear relationship between the average flow of the Cruces River and annual rainfall in Valdivia city [19]. To better observe this, we analyzed the sum of the discharge flows of the Calle-Calle and Cruces rivers between 2008 and 2019 and obtained the trend of their anomalies (Figure 6). For the whole period indicated, there was a negative trend of −96.3 m^3^ s^−1^ per decade^−1^, but when we considered seasons separately these trends changed to −24.6, −113.4, −280.2, and +31.5 m^3^ s^−1^ per decade^−1^ for austral summer, autumn, winter, and spring, respectively. This means that if these trends are maintained over a longer time, saltwater intrusions will potentially happen throughout all seasons, except for austral spring when they will contract.

Furthermore, as can be seen in the sections along the estuary of the Valdivia River (Figure 2), in the position reached by the intrusion of saline water, the water column is almost homogeneous and, therefore, it is not important at what depth the water is extracted. In addition, the situation is more worrying towards the south of Lido, specifically in the sector of Torobayo (Figure 1), where the city of Valdivia has expanded and where there is no drinking water network and the inhabitants are supplied with water from underground sources. Increased salinities across the VRE are expected to intensify saltwater intrusion into groundwater. Furthermore, with more people living there, there also will be more pumping of fresh water from coastal aquifers. This will allow saltwater to enter shallower depths during the dry months. Therefore, to avoid this, it is crucial to maintain the proper balance between the water that is pumped from an aquifer and the amount of water that recharges it. This would not only affect water for human consumption but also for other uses (e.g., agricultural and industrial). An integrated surface water and groundwater modeling system linking salt transport within the VRE with density-dependent groundwater flow in the coastal aquifer may serve as an invaluable management tool to evaluate the overall impact of saltwater intrusion on freshwater resources in the region. This requires continuous monitoring of a network of wells that allows for early warnings of saltwater intrusion and, accordingly, the design of strategies for its control. Finally, it should be added that the construction of the San Pedro hydroelectric plant has been approved [36], which includes a reservoir upstream in the San Pedro River, which flows into the Calle-Calle River and could therefore modify the seasonal variability of saltwater intrusion as it has done in other estuaries, e.g., the San Francisco estuary [37].

## 5. Conclusions

Time series showed that saline intrusions occurred ~20 km from the Valdivia estuary mouth when the total discharges of the Cruces and Calle-Calle rivers was below the threshold of 280–300 m^3^ s^−1^. In addition, when the discharges were lower than the threshold, we observed that the salinities varied with the mixed-semidiurnal tide and the spring–neap and apogee–perigee cycles. Finally, we showed the impact of the discharges of both rivers on the extension of the saline intrusion along the VRE, which fits better to a fractional power model close to −1/3. This model indicates that when the sum of the flows of both rivers is less than 74 m^3^ s^−1^, the saline intrusion arrives at the Cuesta Soto water intake from which the city of Valdivia is supplied during the low rainfall season. Central Chile is going through a mega-drought [38], and projections of the high-emission scenario Representative Concentration Pathway of radiative forcing 8.5 W m^−2^ 2050 indicate that there will be a decrease in rainfall of 15–20% in this region [39]. Therefore, it is expected that saline water intrusions will enter the city water intake more often, causing problems in the city drinking water supply. This implies the need to improve nowcasting and forecasting capabilities through hydrodynamic models that provide different scenarios of saltwater intrusions into the VRE. Obtaining projections and developing numerical experiments under scenarios of global change are currently part of our ongoing research efforts.

## Figures and Tables

**Table 1.** Dates at which hydrographic transects were carried out along the central axes of the VRE.

Season	Date of Sampling	

	Neap Tide	Spring Tide

Austral spring	29/11/2017	06/12/2017
Austral summer	11/01/2018	01/02/2018
Austral fall	08/05/2018	17/05/2018
Austral winter	19/08/2018	12/08/2018

**Table 2.** Correlation coefficients (R^2^) for the regression models that best fitted the relationship between different discharge methods and the extent of saltwater intrusion.

Method	Linear Regression		Power Regression		Exponential	

	Model	R^2^	Model	R^2^	Regression Model	R^2^

Hydrologic flood	y = −0.002895x + 18.30	0.55	y = 66.86x^−1/4.46^	0.69	y = 18.64e^−0.0002x^	0.56
Complete hydrologic flood	y = −0.002227x + 18.13	0.55	y = 63.09x^−1/4.75^	0.70	y = 18.42e^−0.0002x^	0.56
Complete value	y = −0.009129x + 20.79	0.72	y = 163.6x^−1/2.64^	0.88	y = 22.41e^−0.0007x^	0.77
Averaging	y = −0.008321x + 20.28	0.53	y = 117.9x^−1/3.08^	0.53	y = 21.20e^−0.0006x^	0.53
Complete averaging	y = −0.007197x + 19.98	0.64	y = 121.9x^−1/3.00^	0.77	y = 20.98e^−0.0005x^	0.67

**Table 3.** Characteristics of the main harmonic tidal constituents at stations Lido and Valdivia.

Harmonic Constituent	Period (h)	Lido Amplitude * (m)	Lido Greenwich Phase Lag (°)	Lido % Explained	Valdivia Amplitude * (m)	Valdivia Greenwich Phase Lag (°)	Valdivia % Explained

Luni-solar Diurnal (K1)	23.93	0.132	36.51	9.83	0.130	26.50	9.90
Principal lunar Diurnal (O1)	25.82	0.094	357.57	4.97	0.096	349.61	5.40
Principal Lunar Semi-diurnal (M2)	12.42	0.327	41.22	60.45	0.346	30.52	69.80
Principal solar Semi-diurnal (S2)	12.00	0.114	51.75	7.39	0.121	42.71	8.56
Larger lunar elliptic Semi-diurnal (N2)	12.66	0.072	18.85	2.93	0.073	7.81	3.15

* The sums of the amplitude of the main harmonic tidal constituents for Lido and Valdivia stations were 0.74 and 0.77 m, respectively.

**Table 4.** Monthly mean and standard deviation of the wind magnitude (m s^−1^) for the months of lowest discharge at the weather station located in Niebla (Figure 1, green square).

Year	January		February		March		April	

	Mean	Standard Deviation	Mean	Standard Deviation	Mean	Standard Deviation	Mean	Standard Deviation

2018	1.89	0.54	1.78	0.70	1.81	0.70	*	*
2019	1.91	0.78	2.02	0.76	1.89	0.92	1.75	0.81

* The values for April 2018 were not considered because the total average discharge of the rivers was greater than 400 m^3^ s^−1^.
